# Association between chronic pain medications and the severity and mortality of COVID-19: Study protocol for a case-population study

**DOI:** 10.1097/MD.0000000000026725

**Published:** 2021-07-30

**Authors:** Andrés Ancor Serrano Afonso, Concepción Pérez Hernández, Dolores Ochoa Mazarro, Manuel Román Martínez, Inmaculada Failde Martínez, Antonio Montes Pérez, Pablo López Pais, Luz Cánovas Martínez, Miren Revuelta Rizo, María Luz Padilla del Rey, Ana Peiró Perió, Teresa Aberasturi Fueyo, César Margarit Ferrí, Elena Rojo Rodríguez, Agustín Mendiola de la Osa, Manuel José Muñoz Martinez, María Jesús Domínguez Bronchal, Manuel Herrero Trujillano, José Cid Calzada, Gustavo Fabregat-Cid, María José Hernández-Cádiz, Manuel Mareque Ortega, Leticia Gómez-Caro Álvarez Palencia, Víctor Mayoral Rojals

**Affiliations:** a Pain Unit, Anthesthesiology Department, Hospital Universitari de Bellvitge - IDIBELL, L’Hospitalet de Llobregat, Barcelona, Spain; b Pain Unit, Anthesthesiology Department, Hospital Universitario de la Princesa, Madrid, Spain; c Department of Clinical Pharmacology, Hospital Universitario de la Princesa, Madrid, Spain; d Department of Preventive Medicine and Public Health, Universidad de Cádiz, Cadiz, Spain; e Pain Unit, Anthesthesiology Department, Hospital del Mar-IMIM, Barcelona, Spain; f Pain Unit, Anthesthesiology Department, Complejo Hospitalario Universitario de Santiago, Santiago de Compostela, Spain; g Pain Unit, Anthesthesiology Department, Complejo Hospitalario Universitario de Ourense, Ourense, Spain; h Pain Unit, Anthesthesiology Department, Hospital de la Santa Creu i Sant Pau, Barcelona, Spain; i Pain Unit, Anthesthesiology Department, Complejo Hospitalario Universitario de Cartagena, Murcia Spain; j Pain Unit, Anthesthesiology Department, Hospital General Universitario de Alicante, Alicante, Spain; k Pain Unit, Anthesthesiology Department, Hospital Comarcal Alt Penedes, Barcelona, Spain; l Pain Unit, Anthesthesiology Department, Hospital Puerta de Hierro, Madrid, Spain; m Pain Unit, Anthesthesiology Department, Complejo Hospitalario Universitario de Toledo, Toledo, Spain; n Pain Unit, Anthesthesiology Department, Consorcio Hospital General Universitario de Valencia, Valencia, Spain.

**Keywords:** analgesics, chronic pain, coronavirus disease 2019, glucocorticoids, non-steroidal anti-inflammatory drugs, opioids

## Abstract

In patients with coronavirus disease 2019 (COVID-19) infection, common drugs may exacerbate symptoms and negatively impact outcomes. However, the role of chronic medications on COVID-19 effects remains poorly understood. We hypothesized that certain chronic pain medications would influence outcomes in patients with COVID-19.

The main aim is to assess the effect of these medications on the course of the disease in COVID-19 patients. Secondary aims are to compare disease severity and outcomes in patients with COVID-19 receiving chronic treatment with analgesics or other medications versus untreated patients and to determine prevalence of chronic pain medications in specific subgroups of hospitalized patients for COVID-19.

Multicenter case-population study in 15 care centers for patients ≥18 years of age diagnosed and hospitalized with COVID-19. Controls will include patients treated at participating centers for chronic pain during the six-month period prior to March 15th, 2020. Each case will be age- and sex-matched to 10 controls. Patients will be grouped according to disease severity criteria. The primary outcome measures in patients admitted for COVID-19 will be:

1. statistical association between chronic pain medication and disease severity;
2. association between chronic pain treatment and survival.

Secondary outcome measures include:

1. prevalence of chronic pain medications in patients with COVID-19 by age and sex;
2. prevalence of chronic pain medications in patients with COVID-19 vs controls.

Patients and controls will be paired by age, sex, and geographic residence. Odds ratios with 95% confidence intervals will be calculated to determine the association between each drug and clinical status. Univariate and multivariate analyses will be performed.

This is a study protocol. Data is actually being gathered and results are yet not achieved. There is no numerical data presented, so the conclusions cannot be considered solid at this point.

Pain medications are likely to influence severity of COVID-19 and patient survival. Identifying those medications that are most closely associated with severe COVID-19 will provide clinicians with valuable data to guide treatment and reduce mortality rates and the long-term sequelae of the disease.

## 1 Introduction

In the recent year, numerous studies have been performed to better understand the novel coronavirus SARS-CoV-2, the disease it causes, coronavirus disease 2019 (COVID-19), and other medical problems related to it, particularly the factors associated with the risk of severe disease and mortality.^[1]^ The COVID-19 virus penetrates cells by binding to the angiotensin-converting enzyme type 2 (ACE2) receptor, widely expressed throughout the body, through the spike protein,^[2,3]^ in a manner similar to the coronavirus responsible for the SARS epidemic in 2002 to 2003.^[4]^ It has been hypothesized that severe acute respiratory syndrome coronavirus 2 has a high affinity for this receptor, which allows the virus to enter the cell and replicate. ACE2 is a homologue of the angiotensin-converting enzyme (ACE), which plays a key role in regulating blood pressure by converting angiotensin I to angiotensin II, which acts on the angiotensin II type 1 (AT_1_) receptor to produce vasoconstriction.^[5]^ In addition, angiotensin II increases production of aldosterone to promote the reabsorption of sodium and water. This can result in an increase of blood pressure.^[6]^ In contrast, ACE2 inactivates angiotensin II, leading to an increase in angiotensin 1–7 (Ang 1—7), a peptide with a potent vasodilator action. This increase of Ang 1—7 serves as a negative regulator of the renin-angiotensin-aldosterone system (RAAS).^[7]^ But this is not the only action of angiotensin 1–7. It also has anti-inflammatory properties. In addition, several other groups of drugs have been shown to block RAAS, primarily ACE inhibitors (i.e., enalapril or fosinopril), angiotensin II receptor blockers (ARB) (i.e., candesartan or valsartan), and anti-aldosterone agents (i.e., spironolactone or eplerenone). These drugs have all been shown to increase ACE2 expression,^[6]^ which could facilitate viral entry into the targeted cells.^[8]^

The available evidence indicates that certain factors—primarily older age and the presence of pre-existing medical conditions such as hypertension, diabetes, and ischemic heart disease—are the main variables associated with poor outcomes in patients with COVID-19.^[9]^ These conditions are associated with RAAS imbalances, even in the absence of an active medication that directly acts on RAAS. Importantly, these pre-existing comorbidities are more prevalent in older people, who are also more likely to be taking medications—often in polypharmacy—such as thiazolidinediones, non-steroidal anti-inflammatory drugs (NSAIDs), ACE inhibitors, ARBs, and corticosteroids. Indeed, several studies suggest that some of these drugs may exacerbate symptoms of COVID-19, leading to worse outcomes.^[10–12]^

These aggravating factors are all associated with the use of RAAS blockers, which—together with the increased expression of ACE2 induced by these drugs—suggests that the use of RAAS blockers (particularly ACE inhibitors) could be a risk factor for disease severity and poor prognosis in patients with COVID-19.^[10,11]^ Nonetheless, there is some evidence to suggest that ARBs could protect against lung injury in COVID-19.^[8]^ Clearly, there are many open questions and controversies surrounding the effect of these and other medications on the course of disease in patients with COVID-19.^[11]^

The AT_1_ receptor mediates most of the activity of angiotensin II type 2 (AT_2_) and is implicated in multiple pathways related to stress response regulation. Stimulation of the AT_1_ receptor contributes to the release of different types of markers. For instance, AT_2_ interacts with the AT_1_ receptor, activating the NADPH-oxidase complex, the microglial complex via RhoA/Rho kinase, nuclear factor kappa B, inducible nitric oxide synthase, and cyclooxygenase-2. In turn, activated cyclooxygenase-2 produces central and peripheral inflammation as well as oxidative and nitrosative stress.^[13]^ Stimulation of the AT_1_ receptor also releases tumor necrosis factor, which plays an important role in various neurodegenerative disorders and different pain disorders and also regulates activation of the hypothalamic-pituitary-adrenal axis (HPA).^[14]^

Stimulation of the AT_1_ receptor in the hypothalamus increases production of corticotropin-releasing hormone, which in turn stimulates secretion of adrenocorticotropic hormone in the anterior pituitary gland, initiating the stress response cascade. Consequently, in humans, blockade of the AT_1_ receptor activates the HPA pathway.^[14]^ The role of angiotensin II receptors in the production of cytokines and proteoglycans has led to numerous warning about the use of NSAIDs (particularly ibuprofen) in patients with COVID-19, although the potential negative effects of these drugs have not been confirmed by the European Medicines Agency.^[15]^ Similarly, the effects of corticosteroids also remain unclear.^[16]^ By contrast, the role of interleukin 6 (IL-6) in the production of cytokines, especially in patients with severe COVID-19, has been well-established. In fact, the IL6 receptor inhibitor tocilizumab has been used extensively to treat these patients. Relevantly, many drugs routinely used for chronic pain management have been shown to decrease IL-6 levels, especially lidocaine IV, cannabinoids, and (indirectly) anticonvulsants.^[17–26]^

According to the 2017 National Health Survey in Spain,^[27]^ low back pain and osteoarthritis are the second and third most common health problems in Spain, affecting 18.5% and 17.5%, respectively, of the population. Given this high prevalence, the potential effects of drugs used to treat these and other comorbidities in patients with COVID-19 may be particularly relevant for individuals receiving medical treatment for chronic pain, which include NSAIDs, paracetamol, metamizole, corticosteroids, opioids, anticonvulsants, antidepressants, lidocaine, ketamine, cannabinoids, and naltrexone. However, some of these medications may actually have a beneficial effect; for example, intravenous lidocaine^[17,26]^ and cannabinoids may reduce IL-6, an inflammatory cytokine that is often significantly elevated in patients with COVID-19, particularly in severe cases.^[28]^ By contrast, other analgesics, particularly those that produce pulmonary vasoconstriction, such as opioids (e.g., buprenorphine and tramadol) and antidepressants that inhibit serotonin reuptake, could negatively impact the course of COVID-19.^[29]^ Similarly, the effect of other opioids widely used to treat chronic pain are also unknown and may have contradictory effects on the lung and possibly on the effects of ARBs (e.g., serotonin reuptake inhibition, decrease in the HPA axis, and immunomodulator effects on interleukins).^[25,26,30–38]^ Regrettably, at present, the potential effects of most of these chronic pain medications on the clinical course of patients with COVID-19 are not well-understood.^[39]^ Given the severity of COVID-19, particularly among older people and those with pre-existing conditions, there is an urgent need to determine whether and how these commonly used drugs influence outcomes of this novel disease.

The main aim of this study is to assess the effect of the aforementioned medications on the course of the disease in COVID-19 patients. Moreover, we also want to compare disease severity and outcomes in patients with COVID-19 receiving chronic treatment with analgesics or other medications before acquiring the disease, with untreated patients. Furthermore, another aim is to determine the prevalence of chronic pain medications in specific subgroups of hospitalized patients for COVID-19.

## 2 Methods

### 2.1 Hypothesis

We hypothesized that certain analgesics and other chronic pain medications (see Supplemental Digital Content - Annex S1) could influence disease severity and mortality in patients with COVID-19. To assess the influence of these drugs, we grouped patients into different categories according to their clinical status (severity criteria):

1. hospital admission without need for oxygen therapy
2. admission requiring oxygen therapy support alone,
3. admission (inpatient ward or emergency department) requiring non-invasive ventilation;
4. admission to intensive care unit requiring mechanical ventilation (supine, prone, or extracorporeal membrane oxygenation) and
5. death due to COVID-19.

#### 2.1.1 Main objective

To determine whether the use of analgesics and chronic pain medications influence the course of disease in patients with COVID-19 based on severity criteria (groups 3, 4, and 5).

#### 2.1.2 Secondary objectives

a) To evaluate survival in patients with COVID-19 receiving chronic treatment with analgesics and other medications versus patients with COVID-19 not taking those drugs. To identify the factors associated with survival in these patients.
b) To determine whether the use of chronic pain medications in patients hospitalized for COVID-19 is greater in specific subgroups (age and sex).
c) To compare the prevalence of these drugs in patients admitted for COVID-19 vs sex- and age- matched controls (patients with chronic pain receiving treatment at the hospital pain management units).

*Design:* This is a case-population study.

This multicenter study involves 15 tertiary care hospitals in Spain (Table 1).

**Table 1:** Participating hospitals.

Hospital name	Abbreviation
1) Hospital Universitario Bellvitge	HUB
2) Hospital Universitario La Princesa	HLPR
3) Complejo Hospitalario de Toledo	CHUT
4) Hospital Comarcal L’Alt Penedes	HCAP
5) Hospital HM Madrid	HMM
6) Hospital Universitario y Politécnico La Fe de Valencia	HUYPLF
7) Puerta de Hierro-Majadahonda	HPH
8) Hospital Clínico de Santiago de Compostela	CHUS
9) Consorcio Hospital General de Valencia	CHGUV
10) Hospital Universitario de Ourense	CHUO
11) Hospital General Universitario Santa Lucía	HGUSL
12) Hospital Universitario de la Santa Cruz i Sant Pau	HUSCSP
13) Hospital General Universitario de Alicante	HGUA
14) Hospital del Mar de Barcelona	HMar

### 2.2 Participants and eligibility criteria

PHASE 1: SELECTION OF CASES

*Participants*: Adult patients (≥18 years) admitted to one of the participating hospitals with a diagnosis of COVID-19 based on a positive reverse-transcription polymerase chain reaction test.

*Study period for phase 1*: The starting date is pretended to be March 15, 2020. The study finalization date may be extended depending on the number of cases. Recruitment may take more than 12 months.

PHASE 2. SELECTION OF CONTROLS

Controls will be obtained from the database of the individual pain management units at each participating hospital. Controls will be selected from patients treated at these pain management units in the 6 month period prior to the official onset of the pandemic (declared by the World Health Organization on March 11, 2020). Controls will be age- and sex-matched to the hospitalized patients diagnosed with COVID-19. All controls will be classified as COVID-19 positive, negative, or status unknown.

### 2.3 Recruitment of cases and controls

Data will be obtained consecutively from all patients admitted to the participating hospitals for COVID-19. Each case will be matched to 10 controls. The sample size will vary based on the prevalence of a given drug (alpha error = 0.05, beta error = 0.20) in the sample according to the data in Table 2.

**Table 2:** Formula to match cases and controls based on prevalence rates for given drugs.

Odds ratio	Prevalence of use of a given drug (%)	Cases, n	Controls, n
≥1.5	5	918	9172
	10	499	4987
	15	363	3624
	20	298	2973
	30	240	2396
≥2.0	5	277	1763
	10	154	1540
	15	115	1146
	20	97	962
	30	81	809

### 2.4 Primary outcome measures

1. The statistical association between chronic pain medication usage (analgesics and adjuvant medications) and disease severity (according to the aforementioned group classification).
2. The association between chronic pain treatment (analgesics and adjuvant medications) and survival risk for patients admitted for COVID-19.

### 2.5 Secondary outcomes

- To verify whether the use of chronic pain medications in patients hospitalized for COVID-19 is greater in specific subgroups (age and sex).
- To identify the prevalence of use of certain analgesics and other chronic medications in patients with COVID-19.
  ∘ In Phase I of this study, we will examine the prevalence of these medications in the patients admitted for COVID-19.
  ∘ In Phase II, we will compare the prevalence of use of each individual medication (analgesics and non-analgesics) in patients admitted for COVID-19 and controls. If a sufficient number of controls are COVID-19+, we will perform a sub analysis to compare patients to COVID-19+ controls.

### 2.6 Data collection for phases I and II

Each patient's medical record number will be obtained and searched for the required data (Supplemental Digital Content - Annex S2). All relevant study variables will be extracted from these medical records and recorded in an electronic case report form (electronic case report form; Microsoft Excel; see Supplemental Digital Content Annex S3) for complete list of variables and codes). Patients will be identified by the hospital acronym chosen by local investigators (e.g., Hospital La Princesa - HLPR) and assigned a consecutive number. The data collected (de-identified) will be sent on a weekly basis to the lead investigator as pseudo anonymized data in accordance with the recommendations of the International Conference of Harmonization for Good Clinical Practice. All data will be verified and validated by a biostatistician at the University of Cádiz. The Clinical Pharmacology Unit at the HLPR will conduct quality control measures to ensure data integrity.

### 2.7 Statistical analysis

We will perform both univariate and multivariate analyses. For the univariate analysis, a descriptive analysis of the data will be carried out to show the distribution of the qualitative variables as absolute (n) and relative frequencies (%) and central tendency (mean) and dispersion (standard deviation) for quantitative variables. The Kolmogorov-Smirnov test will be applied to check distribution normality of the quantitative variables.

We will calculate the proportion of patients with COVID-19 receiving any of the following medications prior to hospital admission (index date): NSAIDs, corticosteroids, opioids, anticonvulsants, antidepressants, lidocaine, and cannabinoids (together and separately). These values will then be compared to the exposure to these drugs in a random sample of controls treated in the pain management units of the participating hospital during the 6 months prior to study initiation. Patients and controls will be paired by age, sex, and residence (regions).

The crude odds ratio and 95% confidence intervals will be calculated to determine the association between each drug and the patient's clinical status based on the severity criteria of the aforementioned groups:

1. admission (inpatient ward or emergency department) requiring non-invasive ventilation;
2. admission to intensive care unit requiring mechanical ventilation (supine, prone, or extracorporeal membrane oxygenation,
3. death due to COVID-19, and
4. a single variable that combines the 3 previous variables.

A multivariate analysis will be performed and, for each of the variables, a conditioned logistic regression model will be constructed and the adjusted odds ratio for comorbidities and other treatments will be estimated. We will assess whether sex and age act as effect modifiers.

Survival in patients hospitalized for COVID-19 will be analyzed using the Kaplan–Meier method. The start and end dates will be symptom onset (start) and the outcome (death or discharge) as the end date of the study (only for Phase 1).

To identify the factors associated with overall survival time, an adjusted Cox regression model will be performed with hazard ratios (HR) and 95% confidence intervals for each of the pharmacological groups for the treatment of pain and adjuvants, and others factors of known clinical relevance for the survival of COVID-19 patients.

The statistical analyzes will be performed with the IBM-SPSS statistical software package, v.24.0 (IBM-SPSS, Chicago, IL).

In phase 1, intermediate analyses will be performed as the total number of patients increases (e.g., at 100, 250, and 500 cases). At each point, we will collect data for the matching controls. The Scientific Committee will assess the need to continue or not with data collection at each of these intermediate points. Ethics Committee approval, if applicable, will be requested at that time.

## 3 Results

This is a study protocol. Data is actually being gathered and results are yet not achieved. There is no numerical data presented, so the conclusions cannot be considered solid at this point. We intend to get full data in late 2021 and present results on congresses and publish them in a peer reviewed journal, independently of what we find.

## 4 Discussion

It is highly likely that certain analgesics and other drugs used to treat chronic pain could influence the prevalence and severity of COVID-19. Data on the potential influence of most of these drugs in patients with COVID-19 is lacking, yet urgently needed.^[35]^ Several medical societies have published recommendations on pain treatments in these patients^[12,39–45]^ —mainly NSAIDs, corticosteroids, and opioids— but recommendations for the use of other pain treatments are scant.^[40,44,45]^ The rationale underlying these recommendations for limiting the use of all of the aforementioned drugs is based on the immunosuppressive effects that these drugs trigger.^[39,40,43,45,46]^. Immunosuppression can increase susceptibility to secondary infections, although these depends on the specific opioid, as the effects on the immune system can vary.^[47]^ However, at present, there is no evidence regarding the effects of opioids on COVID-19, and pain per se is also a well-known immunosuppressive agent.

Disentangling the specific contribution of individual medications on the clinical course of patients with COVID-19 is highly challenging due to the complex interplay between these agents and other variables (i.e., disease stage, patient-specific variables, treatments, etc.). Another confounding factor is that some therapies used to treat patients with COVID-19, such as hydroxychloroquine^[48]^ or lopinavir/ritonavir,^[49]^ can be neurotoxic while others, such as azithromycin^[50]^ or tocilizumab,^[51]^ among others, have neuroprotective properties.

Given the potential severity of this disease, particularly in patients with pre-existing conditions, together with the likelihood that the COVID-19 pandemic seems likely to be with us for an extended period of time, with ongoing outbreaks,^[52]^ it is crucial that we make every effort to identify the medications most likely to negatively impact the clinical course in these patients. Particularly when the scope of COVID-19 being an endemic virus increases over time and with new strains appearing over and over.

In the present study, we expect to identify those medications that are most closely associated with severe COVID-19. If successful, these findings will provide clinicians with valuable data to help guide and improve treatment of COVID-19 in the near future, potentially reducing mortality rates and reducing the long-term sequelae of the disease. We also expect to help clinicians attending chronic pain patients to be able to choose between those drugs, so that the likelihood of an increase in severity of SARS-CoV-2, after getting infected with COVID-19, is diminished. These data may also provide clinicians with a tool to treat pain patients in the context of the pandemic until an effective treatment or vaccine for COVID-19 has been obtained.

### 4.1 Limitations

As the knowledge of the COVID-19 has been increasing through time, treatment strategies have been continuously developing and changing. As a consequence, study results can be biased. Also, there will be limitations on the control group on phase 2. Undoubtedly, the results are to be conditioned to the time when the study is done, and the characteristics of the patients admitted at that time. As the epidemiological situation is changing very quickly and differs from country to country, a study on a different period of time or country could not achieve the same results.

At the beginning of the pandemic the available COVID-19 tests were scarce. So there is the possibility that we can count control group populations that were on COVID-19, but asymptomatic, or even mildly symptomatic but not enough even to go to the emergency room. We know that this is a main limitation and confusion factor for the study. Another limitation, or confusion factor would be overestimating impact of severity. As we are looking only on patients admitted to hospital, we can be overestimating the impact of such drugs. Finally, as this study has not received any specific grant nor funding, and, thus, data is going to be gathered by authors on their available spare free time, there is the likelihood that data captures are not finished in time, or that we are not able to include a big amount of control group population. This limitation also applies to the possibility that the number of patients included on each site are not proportional to the hospital or the population corresponding to the site, but to the spare free time of the site team.

## 5 Conclusion

In the present study protocol, we expect to be able to identify those medications that are most closely associated with severe COVID-19. If successful, these findings will provide clinicians with valuable data to help guide and improve treatment of COVID-19 in the near future, potentially reducing mortality rates and decreasing the long-term sequelae of the disease. We also expect to help managing chronic pain patients avoiding those drugs that could increase severe acute respiratory syndrome coronavirus 2 severity. These data may also provide clinicians with a tool to treat pain patients in the context of the pandemic until an effective treatment or vaccine for COVID-19 has been obtained.

## Acknowledgments

The authors would like to thank Bradley Londres for professional English language editing. We thank CERCA Programme / Generalitat de Catalunya for institutional support.

## Author contributions

**Conceptualization:** Dolores Ochoa Mazarro, Manuel Román Martínez, Inmaculada Failde Martínez, Teresa Aberasturi Fueyo, Agustí Mendiola de la Osa, Víctor Mayoral Rojals.

**Data curation:** Ancor Serrano Afonso, Manuel Román Martínez, Antonio Montes Pérez, Pablo López Pais, Luz Cánovas Martínez, Miren Revuelta Rizo, María Luz Padilla del Rey, Ana Peiró Perió, Teresa Aberasturi Fueyo, César Margarit Ferrí, Elena Rojo Rodríguez, Agustí Mendiola de la Osa, Manuel José Muñoz Martínez, María Jesús Domínguez Bronchal, Manuel Herrero Trujillano, Gustavo Fabregat-Cid, María José Hernández-Cádiz, Manuel Mareque Ortega, Leticia Gómez-Caro Álvarez Palencia, Víctor Mayoral Rojals.

**Formal analysis:** Dolores Ochoa Mazarro, Manuel Román Martínez, Inmaculada Failde Martínez, Antonio Montes Pérez, Agustí Mendiola de la Osa, Víctor Mayoral Rojals.

**Investigation:** Ancor Serrano Afonso, Concepción Pérez Hernández, Dolores Ochoa Mazarro, Antonio Montes Pérez, Luz Cánovas Martínez, Miren Revuelta Rizo, María Luz Padilla del Rey, Ana Peiró Perió, Teresa Aberasturi Fueyo, Elena Rojo Rodríguez, Manuel José Muñoz Martínez, María Jesús Domínguez Bronchal, Manuel Herrero Trujillano, José Cid Calzada, Gustavo Fabregat-Cid, María José Hernández-Cádiz, Leticia Gómez-Caro Álvarez Palencia, Víctor Mayoral Rojals.

**Methodology:** Ancor Serrano Afonso, Concepción Pérez Hernández, Dolores Ochoa Mazarro, Manuel Román Martínez, Elena Rojo Rodríguez, Manuel Herrero Trujillano, Gustavo Fabregat-Cid, Víctor Mayoral Rojals.

**Project administration:** Víctor Mayoral Rojals.

**Resources:** Ancor Serrano Afonso, César Margarit Ferrí, Manuel José Muñoz Martínez, María José Hernández-Cádiz, Manuel Mareque Ortega, Leticia Gómez-Caro Álvarez Palencia, Víctor Mayoral Rojals.

**Supervision:** Ancor Serrano Afonso, Concepción Pérez Hernández, Inmaculada Failde Martínez, Manuel José Muñoz Martínez, Víctor Mayoral Rojals.

**Validation:** Ancor Serrano Afonso, Dolores Ochoa Mazarro, Manuel Mareque Ortega, Víctor Mayoral Rojals.

**Visualization:** Ancor Serrano Afonso, Elena Rojo Rodríguez, Víctor Mayoral Rojals.

**Writing – original draft:** Ancor Serrano Afonso, Inmaculada Failde Martínez.

**Writing – review & editing:** Ancor Serrano Afonso, Concepción Pérez Hernández, Manuel Román Martínez, Inmaculada Failde Martínez, Antonio Montes Pérez, Pablo López Pais, Ana Peiró Perió, Víctor Mayoral Rojals, María Luz Padilla del Rey.

## Supplementary Material

Supplemental Digital Content
