# Supplementary material for: Association between chronic pain medications and the severity and mortality of COVID-19: Study protocol for a case-population study
Source: Medicine (Baltimore). 2021 Jul 30;100(30):e26725. doi: 10.1097/MD.0000000000026725 (PMC8322492; doi:10.1097/MD.0000000000026725)
Supplement: Supplemental Digital Content [file medi-100-e26725-s001.docx]

**Supplementary Digital Content**

**Annex I: Sociodemographic, comorbidities, and adjuvant variables**

| **VARIABLES** | **CODE** | **TYPE** | **VALUES** | **OBSERVATIONS** |
| --- | --- | --- | --- | --- |
| Code | Id | Quantitative | 1-n | - |
| MRN (medical record number), recoded for confidentiality | MRN | - | - | A cross-matched list will be created with the identification code and this will be removed from the working database |
| Hospital | Hospital | Nominal | - | Initials of treating hospital |
| Data of birth | - | Date | dd/mm/yyyy | - |
| Age at diagnosis | Edad | Quantitative | 0-n | years |
| Sex | Sex0 | Nominal | 1: Male  2: Female | - |
| Race, Caucasian | Raza | Nominal | 1: Yes  2: No | - |
| Date of admission | Fecha ing | Date | dd/mm/yyyy | - |
| Course after 1^st^ visit to emergency department | Evolución | Nominal | 1: Hospital admission  2: Transfer to other hospital | - |
| Date of symptom onset | Date_ini_sint | Date | dd/mm/yyyy | - |
| Date of transfer to other hospital | Date_trasl | Date | dd/mm/yyyy | - |
| The patient could not be transferred to the ICU due to lack of means, but met clinical criteria. | uci_criterio | Nominal | 1: Si  2: No | - |
| Date of diagnosis | Date_dx | Date | dd/mm/yyyy | - |
| ICU | uci | Nominal | 0:no  1: Yes | Patient admitted to ICU |
| Date of admission to ICU | Date_uci | Date | dd/mm/yyyy | - |
| Outcome | desenlace | Nominal | 0: unresolved  1: Discharged  2: Death | - |
| Date of outcome | Date_desenlac | Date | dd/mm/yyyy | - |
| History of arterial hypertension | hta | Nominal | 0:no  1: Yes | - |
| History of chronic pain | Dolor Crónico | Nominal | 0: No  1: Yes | - |
| Type of pain | Tipo de dolor | Nominal | Neuropathic  Somatic  Visceral | - |
| History of diabetes | diabetes | Nominal | 0:no  1: Yes | Type 1 or 2 |
| Obesity | obes | Nominal | 0:no  1: Yes | Defined as BMI>30kg/m^2^ |
| History of dyslipidemia | dislip | Nominal | 0: no  1: Yes | - |
| History of hyperuricemia | Hiperuricemia | Nominal | 0: no  1: Yes | - |
| History of acute myocardial infarction/ angina/ ischemic cardiopathy | iam | Nominal | 0:no  1: Yes | - |
| History of cardiac failure | insuf | Nominal | 0:no  1: Yes | - |
| History of atrial fibrillation | fa | Nominal | 0:no  1: Yes | - |
| History of deep venous thrombosis (DVT)/ pulmonary embolism (PE) | tep_tev | Nominal | 0:no  1: Yes | - |
| History of ictus | ictus | Nominal | 0:no  1: Yes | - |
| History of COPD | epoc | Nominal | 0:no  1: Yes | - |
| History of asthma | asma | Nominal | 0:no  1: Yes | - |
| Previous cancer | cancer_prev | Nominal | 0:no  1: Yes | - |
| Active cancer | cancer_act | Nominal | 0:no  1: Yes | - |
| History of chronic renal failure | irc | Nominal | 0:no  1: Yes | - |
| Other illness (e.g., immune system or others) | enf_otras | Nominal | Text chain | - |
| ACE inhibitors | ieca | Nominal | 0: No  1: Yes | Complete if patient received in the month prior to diagnosis |
| ACE inhibitors, active ingredient | ieca_pa | Nominal | Text chain | - |
| ACE daily dose | ieca_dd | Quantitative | 0-n | Dose in mg/day |
| Angiotensin receptor blockers (ARBs) | araii | Nominal | 0: No  1: Yes | Complete if patient received in the month prior to diagnosis |
| ARBs, active ingredient | araii_pa | Nominal | Text chain | - |
| ARBs, daily dose | araii_dd | Quantitative | 0-n | Dose in mg/day |
| Anti-aldosterone | antiald | Nominal | 0: No  1: Yes | Complete if patient received in the month prior to diagnosis |
| Anti-aldosterone, active ingredient | antiald_pa | Nominal | Text chain | - |
| Anti-aldosterone, daily dose | antiald_dd | Quantitative | 0-n | Dose in mg/day |
| Calcium channel blockers (CCB), including diltiazem and verapamil | aca | Nominal | 0: No  1: Yes | Complete if patient received in the month prior to diagnosis |
| CCB, active ingredient | aca_pa | Nominal | Text chain | - |
| Diuretics | diur | Nominal | 0: No  1: Yes: | Complete if patient received in the month prior to diagnosis |
| Diuretics, active ingredient | diur_pa | Nominal | Text chain | - |
| Oral anticoagulants | aco | Nominal | 0: No  1: Yes | Complete if patient received in the month prior to diagnosis |
| Oral anticoagulants active ingredient | aco_pa | Nominal | Text chain | - |
| Antiplatelet therapy | antiag | Nominal | 0: No  1: Yes | Complete if patient received in the month prior to diagnosis |
| Antiplatelets, active ingredient | antiag_pa | Nominal | Text chain | - |
| Nonsteroidal anti-inflammatories (NSAIDs) | aine | Nominal | 0: No  1: Yes: | Complete if patient received in the month prior to diagnosis |
| NSAIDs, active ingredient | aine_pa | Nominal | Text chain | - |
| NSAIDs, daily dose | aine_dd | Quantitative | 0-n | Dose in mg/day |
| Systemic corticosteroids | cortic | Nominal | 0: No  1: Yes | Complete if patient received in the month prior to diagnosis |
| Corticosteroids, route of administration | Cortic_va | Nominal | Indicate route of administration | - |
| Corticosteroids, active ingredient | cortic_pa | Nominal | Text chain | - |
| Corticosteroids, daily dose | cortic_dd | Quantitative | 0-n | Dose in mg/day |
| Paracetamol | para | Nominal | 0: No  1: Yes | Complete if patient received in the month prior to diagnosis |
| Metamizole | meta | Nominal | 0: No  1: Yes | Complete if patient received in the month prior to diagnosis |
| Statins | estat | Nominal | 0:no  1: Yes | Complete if patient received in the month prior to diagnosis |
| Statin, active ingredient | estat_pa | Nominal | Text chain |  |
| Oral antidiabetics | ado | Nominal | 0:no  1: Yes | Complete if patient received in the month prior to diagnosis |
| Oral antidiabetics, active ingredient (including combinations) | ado_pa | Nominal | Text chain | If combinacions, given poner los active ingredient |
| Insulin | insulina | Nominal | 0:no  1: Yes | Complete if patient received in the month prior to diagnosis |
| Tramadol | Tramadol | Nominal | 0:no  1: Yes | Complete if patient received in the month prior to diagnosis |
| Tapentadol | Tapentadol | Nominal | 0:no  1: Yes | Complete if patient received in the month prior to diagnosis |
| Oxycodone/naloxone | Oxicodona/naloxona | Nominal | 0:no  1: Yes | Complete if patient received in the month prior to diagnosis |
| Fentanyl | fentanilo | Nominal | 0:no  1: Yes | Complete if patient received in the month prior to diagnosis |
| Buprenorphine | buprenorfina | Nominal | 0:no  1: Yes | Complete if patient received in the month prior to diagnosis |
| Morphine | morfina | Nominal | 0:no  1: Yes | Complete if patient received in the month prior to diagnosis |
| Codeine | codeína | Nominal | 0:no  1: Yes | Complete if patient received in the month prior to diagnosis |
| Anticonvulsants | anticonvulsivantes | Nominal | 0:no  1: Yes | Complete if patient received in the month prior to diagnosis |
| Anticonvulsants, active ingredient | anticonvulsivantes_pa | Nominal | 0:no  1: Yes | Complete if patient received in the month prior to diagnosis |
| Antidepressive agents | Antidepresivos | Nominal | 0:no  1: Yes | Complete if patient received in the month prior to diagnosis |
| Antidepressants, active ingredient | Antidepresivos_pa | Nominal | 0:no  1: Yes | Complete if patient received in the month prior to diagnosis |
| Lidocaine | lidocaína | Nominal | 0:no  1: Yes | Complete if patient received in the month prior to diagnosis |
| Vitamin D | Vit D | Nominal | 0:no  1: Yes | Complete if patient received in the month prior to diagnosis |
| oxycodone | oxicodona | Nominal | 0:no  1: Yes | Complete if patient received in the month prior to diagnosis |
| Hydromorphone | Hidromorfona | Nominal | 0:no  1: Yes | Complete if patient received in the month prior to diagnosis |
| Ketamine | Ketamina | Nominal | 0:no  1: Yes | Complete if patient received in the month prior to diagnosis |
| THC | THC | Nominal | 0:no  1: Yes | Complete if patient received in the month prior to diagnosis |
| THC active ingredient | THC_pa | Nominal | Text chain | Complete if patient received in the month prior to diagnosis |
| Biologics | Biologicos | Nominal | 0:no  1: Yes | Complete if patient received in the month prior to diagnosis |
| Biologics_pa | Nominal | Nominal | Text chain | ~~-~~ |
| Fibrates for hypertriglyceridemia | Fibratos por hipertrigliceridemia | Nominal | 0:no  1: Yes | Complete if patient received in the month prior to diagnosis |
| Chemotherapy | Quimioterapia | Nominal | 0:no  1: Yes | Complete if patient received in the month prior to diagnosis |
| Radiotherapy | Radioterapia | Nominal | 0:no  1: Yes | ~~-~~ |
| Transplanted | Trasplantados | Nominal | 0:no  1: Yes | ~~-~~ |
| Immunosuppressive | inmunosupresor | Nominal | 0:no  1: Yes | Complete if patient received in the month prior to diagnosis |
| Immunosuppressive_pa | immunosupresor_pa | Nominal | Text chain | ~~-~~ |
| Ferritin | Ferritina_d7 | Quantitative | 0-n | Indicate worst value in the clinical course |
| Lymphocytes | Linfocitos_d7 | Quantitative | 0-n | Indicate worst value in the clinical course |
| D dimer | D_dimero_d7 | Quantitative | 0-n | Indicate worst value in the clinical course |
| LDH | LDH_d7 | Quantitative | 0-n | Indicate worst value in the clinical course |
| IL-6 | IL6_d7 | Quantitative | 0-n | Indicate worst value in the clinical course |
| Kaletra | Kaletra | Nominal | 0:no  1: Yes | - |
| Hydroxychloroquine sulfate | Dolquine | Nominal | 0:no  1: Yes | - |
| Azithromycin | Azitromicina | Nominal | 0:no  1: Yes | - |
| Remdesivir | Rendesivir | Nominal | 0:no  1: Yes | - |
| Tocilizumab | RoActemra | Nominal | 0:no  1: Yes | - |
| Corticosteroids | Corticoides | Nominal | 0:no  1: Yes | - |
| Anticoagulant | Anticoagulante | Nominal | 0:no  1: Yes | - |
| Other treatments | Otros_ttª | Text | Indicate treatments | - |
| Antibiotics | Otros_Antib | Nominal | 0:no  1: Yes | - |
| Type of ventilation | Tipo de ventilación | Nominal | Indicate type of ventilation | - |
| FiO2 | FiO2 | Quantitative | 0-n | - |
| Prone positioning | PRONO | Nominal | 0:no  1: Yes | - |
| Complications during COVID | Complicaciones durante el COVID | Text | - | - |
| Commentary | Commentary | Text | - | - |
